# Enhancing Whole-Brain Magnetic Field Homogeneity for 3D-Magnetic Resonance Spectroscopic Imaging with a Novel Unified Coil: A Preliminary Study

**DOI:** 10.3390/cancers16061233

**Published:** 2024-03-21

**Authors:** Archana Vadiraj Malagi, Xinqi Li, Na Zhang, Yucen Liu, Yuheng Huang, Fardad Michael Serry, Ziyang Long, Chia-Chi Yang, Yujie Shan, Yubin Cai, Jeremy Zepeda, Nader Binesh, Debiao Li, Hsin-Jung Yang, Hui Han

**Affiliations:** 1 Biomedical Imaging Research Institute, Cedars-Sinai Medical Center, Los Angeles, CA 90048, USA; archana.malagi@cshs.org (A.V.M.); xinqi.li@mdc-berlin.de (X.L.); na.zhang@siat.ac.cn (N.Z.); liuyucen95@outlook.com (Y.L.); chrihuan@iu.edu (Y.H.); fardad.serry@cshs.org (F.M.S.); ziyang.long@cshs.org (Z.L.); chia-chi.yang@cshs.org (C.-C.Y.); shan37@purdue.edu (Y.S.); yubincai@media.mit.edu (Y.C.); jeremy.zepeda@cshs.org (J.Z.); debiao.li@cshs.org (D.L.); 2 Department of Bioengineering, University of California, Los Angeles, CA 90095, USA; 3 Department of Radiology, Weill Medical College of Cornell University, New York, NY 10065, USA; 4 Department of Imaging, Cedars-Sinai Medical Center, Los Angeles, CA 90048, USA; nader.binesh@cshs.org

**Keywords:** 3T, magnetic resonance spectroscopic imaging, shim–RF head coils, array coils, local B_0_ shimming, spherical harmonic (SH)

## Abstract

**Simple Summary:**

Magnetic resonance spectroscopic imaging (MRSI) plays an increasingly important role in the non-invasive diagnosis and treatment planning of gliomas. This study introduces an innovative head coil array with integrated high-order B_0_ shimming capabilities to enhance its clinical utility. The head coil array can effectively correct the main B_0_ field inhomogeneity in the brain, resulting in improved whole-brain coverage and data quality for 3D-MRSI while reducing variations in shimming across different subjects. The ultimate goal is to promote the routine clinical use of whole-brain MRSI.

**Abstract:**

The spectral quality of magnetic resonance spectroscopic imaging (MRSI) can be affected by strong magnetic field inhomogeneities, posing a challenge for 3D-MRSI’s widespread clinical use with standard scanner-equipped 2nd-order shim coils. To overcome this, we designed an empirical unified shim–RF head coil (32-ch RF receive and 51-ch shim) for 3D-MRSI improvement. We compared its shimming performance and 3D-MRSI brain coverages against the standard scanner shim (2nd-order spherical harmonic (SH) shim coils) and integrated parallel reception, excitation, and shimming (iPRES) 32-ch AC/DC head coil. We also simulated a theoretical 3rd-, 4th-, and 5th-order SH shim as a benchmark to assess the UNIfied shim–RF coil (UNIC) improvements. In this preliminary study, the whole-brain coverage was simulated by using B_0_ field maps of twenty-four healthy human subjects (*n* = 24). Our results demonstrated that UNIC substantially improves brain field homogeneity, reducing whole-brain frequency standard deviations by 27% compared to the standard 2nd-order scanner shim and 17% compared to the iPRES shim. Moreover, UNIC enhances whole-brain coverage of 3D-MRSI by up to 34% compared to the standard 2nd-order scanner shim and up to 13% compared to the iPRES shim. UNIC markedly increases coverage in the prefrontal cortex by 147% and 47% and in the medial temporal lobe and temporal pole by 29% and 13%, respectively, at voxel resolutions of 1.4 cc and 0.09 cc for 3D-MRSI. Furthermore, UNIC effectively reduces variations in shim quality and brain coverage among different subjects compared to scanner shim and iPRES shim. Anticipated advancements in higher-order shimming (beyond 6th order) are expected via optimized designs using dimensionality reduction methods.

## 1. Introduction

As an imaging method, magnetic resonance spectroscopic imaging (MRSI) [1,2,3,4,5,6,7] holds substantial potential in supplementing routine anatomical MRI for the monitoring of metabolic alterations associated with various neurological disorders. It provides physio-metabolic information about the tumor environment, significantly influencing glioma diagnosis, grading, and the planning of surgical and treatment strategies. MRSI serves as a biomarker to distinguish high-grade and low-grade gliomas and to precisely localize the active tumor region [8,9,10]. Three dimensionally encoded MRSI methods offer enhanced sensitivity per unit of time and volume for brain lesions [3,10,11]. Presurgical and radiation treatment planning would greatly benefit from full 3D information and, ideally, with isotropic resolution. However, this is challenging to achieve with multi-slice methods. The demand for rapid 3D-MRSI techniques is considerable, with echo-planar spectroscopic imaging (EPSI)-based methods prevalently utilized in clinical settings [11]. These methods facilitate comprehensive presurgical and radiation therapy planning by acquiring whole-brain spectroscopy [8]. However, 3D-MRSI encounters notable limitations in clinical implementation, including the similar spectroscopic profiles of metabolites in different pathophysiologies, low signal-to-noise ratio (SNR), susceptibility artifacts, B_0_ field inhomogeneity, and lengthy scan time, despite its non-invasive nature for tumor diagnosis or grading [8,12,13]. Among these challenges, B_0_ field inhomogeneity and variability in shimming across different subjects and MR platforms present significant technical obstacles to the routine clinical application of whole-brain 3D-MRSI [11].

Glioblastoma, the most prevalent adult brain malignancy, frequently leads to poor outcomes due to its aggressive nature, limited response to radiation and chemotherapy, and a tendency for early, localized recurrence. The difficulty in accurately identifying tumor margins, which may extend into surrounding brain tissue undetected by standard imaging, poses a significant challenge. Whole-brain MRSI has been shown to predict relapse sites post-radiotherapy, enhancing the precision of treatment planning [2,14]. The ability of MRSI to detect tumor infiltration more sensitively and precisely than traditional MR imaging allows for more accurate radiation targeting, potentially increasing survival by focusing on both the primary tumor and high-risk recurrence areas. A pilot study on MRSI-guided dose escalation demonstrates the advantage of 3D whole-brain MRSI, highlighting its potential to improve glioblastoma management by customizing radiation therapy to each patient’s tumor metabolism. This approach aimed to improve disease management while minimizing radiation exposure to healthy tissue [2,14,15,16].

A significant percentage of voxels may be unsuitable for spectrum measurement due to low spectral quality in voxels where B_0_ field inhomogeneity correction is absent in MRSI [17]. Moreover, regions with susceptibility artifacts caused by tissue and air interfaces, if uncorrected, can lead to signal dropout due to intravoxel dephasing in the affected areas. In 3D-MRSI experiments employing standard product B_0_ shimming [11], approximately 60% of the brain yields data of suitable quality, with a spectral linewidth of 13 Hz. However, substantial variability is observed across subjects from different vendors and sites, ranging from 78% down to 37% [11]. B_0_ field inhomogeneity adversely affects lineshapes and spectral quality in critical brain areas such as the prefrontal cortex (PFC), medial temporal lobe (MTL), temporal pole, cerebellum, and brainstem, necessitating improved field homogenization to enhance spectral profiles by minimizing local field variations at high field strengths [18].

Hardware-based B_0_ shimming emerges as the most straightforward approach to rectifying B_0_ inhomogeneity, a prevalent issue in MRI [19]. This challenge is intensified by the complex internal human anatomy, which includes organs, air cavities, bones, and often, metallic implants, complicating the shimming process. Typically, standard MR scanners are equipped with vendor-supplied shim coils within the magnet bore, yet their distant placement from the brain limits their effectiveness. These shim coils generally provide only 1st–2nd-order spherical harmonic (SH) shim fields, inadequate for addressing higher-order field variations at tissue-air interfaces, such as sinuses and ear canals. Recently, the trend towards local shimming has gained momentum.

Innovative approaches have been designed to position shim coils closer to the target organ, enabling higher-order shim field patterns and improved shim performance [17,20,21,22,23,24,25,26]. A notable development in this domain is the development of “integrated parallel reception, excitation, and shimming (iPRES)” or “AC/DC” coils [24,25,26], which have gained interest for their potential seamless integration into clinical workflows. iPRES employs radiofrequency (RF) antennae for active B_0_ shimming, combining B_0_ shimming with existing RF coils to optimize performance while minimizing hardware alterations and maintaining patient comfort.

Despite the advancement of iPRES over conventional scanner 2nd-order shim, its static whole-brain shimming capabilities remain suboptimal compared to desired 3rd-order shimming. The increasing demand for higher-order shimming, especially for comprehensive brain coverage in 3D and simultaneous multi-slice (SMS) acquisitions [27,28], underscores the limitations of iPRES, primarily due to the constrained design of shared shim and RF loops.

Recent developments have concentrated on the creation of novel RF-shim circuit designs, which offer increased flexibility in shim field design. Our proposed UNIfied shim–RF coil (UNIC) concept [29,30,31,32,33,34] integrates a versatile local shim array into a standard RF coil without compromising RF sensitivity. This approach allows for the customization of shim loop designs, and our UNIC body coil (12-ch RF receive and 42-ch shim) prototype has demonstrated superior high-order shimming capabilities in cardiac MR [31], liver multi-parametric MRI [32], and distortion mitigation caused by metal implants [33]. These promising results are currently under publication, which highlights UNIC’s potential in advancing MRI technology [29].

In this study, we designed an empirical UNIC head coil with 32-ch RF receiver and 51-ch shim aimed at enhancing whole-brain 3D-MRSI. Leveraging the success of the UNIC body coil prototype, we utilized this empirical head coil in simulated MRSI brain coverage data from twenty-four healthy human subjects to validate its effectiveness. Our study includes a comparative analysis with a standard scanner shim with 2nd-order SH coils and the iPRES 32-channel AC/DC head coil. To benchmark improvements, we also simulated theoretical 3rd-, 4th-, and 5th-order SH shim coils. This approach allowed us to rigorously assess the impact of the UNIC head coil on brain coverage in 3D-MRSI.

## 2. Materials and Methods

### 2.1. Dataset and Imaging Protocol

Brain B_0_ field maps from twenty-four healthy human subjects (n = 24), publicly available via the Human Connectome Project, were utilized in this study, adhering to all relevant guidelines and regulations [35]. These maps were derived from a dual-echo gradient echo sequence with voxel size: 2.4 × 2.4 × 2 mm^3^, field of view (FOV): 214 × 250 × 120 mm^3^; time of relaxation (TR) = 10ms; time of echo (TE) first echo = 2.00 ms; second echo = 4.46 ms; flip-angle = 15° were acquired on a 3T scanner (Skyra, Siemens, Erlangen, Germany).

### 2.2. Coil Design for Whole-Brain Shimming

**Figure 1:**
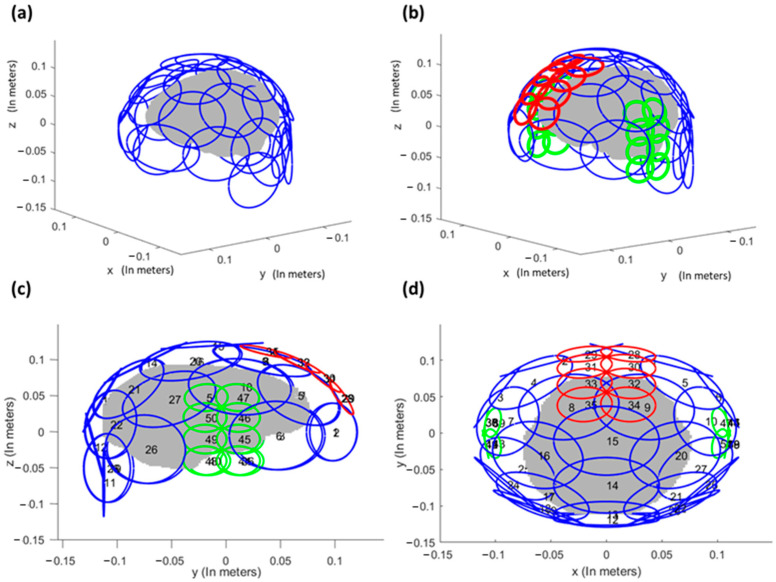
Shim loop layout. (**a**) iPRES 32-ch shim loops (9.5 cm diameter) evenly distributed on the brain surface (gray). (**b**–**d**) UNIC 51-ch shim loop layout. Eight smaller shim loops are arranged on the frontal lobe (red, 5 cm in diameter) and on each ear (green, 4 cm in diameter). The remaining evenly distributed 27 large shim loops are in blue and 9.5 cm in diameter. (**c**) Side-view, and (**d**) Top view. The size-matched shim loops (red and blue) specifically target both the prefrontal and temporal lobes by creating shim fields to counteract the disturbing fields from the nasal cavity and ear, maximizing the shim effectiveness in those regions.

The shim loop layout for the UNIC head coil is illustrated in Figure 1. The empirical coil design builds upon our prior experience with a successfully validated UNIC body coil prototype (currently under publication [29]). Figure 1a shows the traditional 32-ch iPRES shim loop layout with 32 shim–RF shared loops (9.5 cm in diameter) distributed evenly on top of the coil helmet. The UNIC shim loop layout (Figure 1b–d) differs from iPRES in that it includes eight smaller size-matched loops arranged onto the frontal cortex and two temporal lobes, specifically targeting the regions most affected by B_0_ inhomogeneity. Each of these smaller UNIC shim loops consists of 2 turns, effectively doubling the shim field strength per unit current. These 51 UNIC shim loops are overlapped with the 32 RF receive (Rx) loops on the same coil helmet surface. The decoupling scheme between 51 shim loops and 32 RF Rx loops employs the methodology used in our UNIC body coil prototype with 12-ch RF Rx loops and 42-ch shim loops (2 turns), which maintains the RF array receive sensitivity. Key features of the UNIC design include: (i) Allowing size-matched shim loops to be positioned as close as physically feasible to the target structures (e.g., PFC/MTL) to maximize shim effectiveness. (ii) Housing all the shim and RF coils within a standard RF coil assembly, ensuring no patient discomfort and maintaining the simplicity of iPRES.

The process for organizing the loops involves the following steps: Initially, the 32 RF receive loops are evenly dispersed across the helmet surface to cover the entire brain. Subsequently, 8 smaller shim loops are positioned on the frontal brain (red, 5 cm in diameter) and in each ear cavity (green, 4 cm in diameter). Lastly, the remaining 27 large shim loops, each 9.5 cm in diameter and depicted in blue, are evenly distributed. It is worth noting that an alternative implementation of these 27 large shim loops involves using the iPRES shim–RF shared loops. The locations of the shim loops are detailed in the Supplementary Table S1. More details of the UNIC shim loop design and implementation can be found in [29]. However, the absolute mean and maximum currents that were employed across the twenty-four subjects, with currents administered within each 51-channel coil across three representative subjects, are illustrated in Supplementary Tables S2 and S3.

### 2.3. B_0_ Shim Field Analysis and 3D-MRSI Brain Coverage Analysis

To investigate the effectiveness of different B_0_ shim methods for 3D-MRSI acquisitions, B_0_ field maps of the whole brain (60 slices, 2 mm slice thickness) were utilized for B_0_ computation and shim simulation. The field maps were processed with the FSL-Brain Extraction Tool (BET) [36] to extract the brain from magnitude and phase images. Magnetic field simulation was based on the Biot-Savart Law, and such simulations can typically predict experiments accurately [12,19,25]. The proposed UNIC shimming was compared to both 32-ch iPRES shimming and the standard scanner shim using 2nd-order SH shim coils. Theoretical 3rd-, 4th-, and 5th-order SH shims were also simulated as benchmarks to assess the UNIC improvements. Note that these higher-order shim coils are usually not equipped with clinical scanners due to physical limitations. Shim fields from different methods were applied to minimize the absolute means square of the off-resonance field of the whole-brain volume using the linear least-square optimization (“lsqlin”) in MATLAB (Mathworks, Natick, MA, USA). DC currents of the surface shim coils were constrained at ±3.0 A per coil. Following the B_0_ shimming, the whole-brain field homogeneity (standard deviation (SD) of off-resonance frequency) and MRSI coverage were derived.

To quantify the MRSI coverage of the brains, the spectral linewidth, $∆\nu_{line\;width}\;$ was calculated for each voxel using Equation (1) [37]:

(1) $$∆\nu_{line\;width}=\frac{1}{\pi *T2}+∆\nu^{\prime }+∆\nu$$

where $∆\nu_{line\;width}$ is dependent on *T*2, $∆\nu^{\prime }$ is microscopic susceptibility, and ∆ν is macroscopic susceptibility term. In past literature, it was shown that $\frac{1}{\pi *T2}+∆\nu^{\prime }$ can be less than 10 Hz, and we assumed 9 Hz for this study [37]. The macroscopic susceptibility term was measured using a rescaled B_0_ field map and transformed into linewidth space. This transformation into linewidth was achieved at varying spatial resolution by calculating the standard deviation (SD) for all neighboring voxels. For example, for an MRSI scan with a spatial resolution of 12 × 12 × 10 mm^3^ and a 1.44 cubic centimeter (cc) voxel size, each voxel comprises 125 pixels from a B_0_ field map with a resolution of 2.4 × 2.4 × 2 mm^3^. The SD of the voxel was calculated based on these 125 voxel values. As per the previous studies, only voxels with linewidth less than 18 Hz were considered to match the water spectra linewidth and create a mask [11,37]. To calculate the total brain coverage in percent, the number of voxels in the mask was divided by the total number of voxels in the brain before thresholding.

Region-based brain coverage was evaluated by segmenting certain brain regions that are prone to B_0_ field inhomogeneity. Two ROIs were drawn in the frontal lobe, which included the prefrontal cortex (PFC), and another in the temporal lobe, which included both the medial temporal lobe and the temporal pole (MTL + temporal pole). ROIs were drawn manually using the Harvard–Oxford cortical and subcortical structural atlas as a reference [38].

### 2.4. Statistical Analysis

One-way analysis of variance (ANOVA) was used to investigate significant differences between the SD of B_0_ field and brain coverage of MRSI with different shimming techniques. All pairwise comparisons were adjusted for multiple comparisons using the Bonferroni test, and a *p*-value of <0.05 was considered significant. Data are represented as mean ± SD. Data from the whole brain and specific anatomical areas, such as the frontal lobe, PFC, temporal lobe, MTL, and temporal pole, were used in all analyses. All the statistical analyses were performed using SPSS software (SPSS version 24, Chicago, IL, USA).

## 3. Results

### 3.1. Whole-Brain B_0_ Shimming Performance of Spherical Harmonic (SH), iPRES, and UNIC Shim

**Figure 2:**
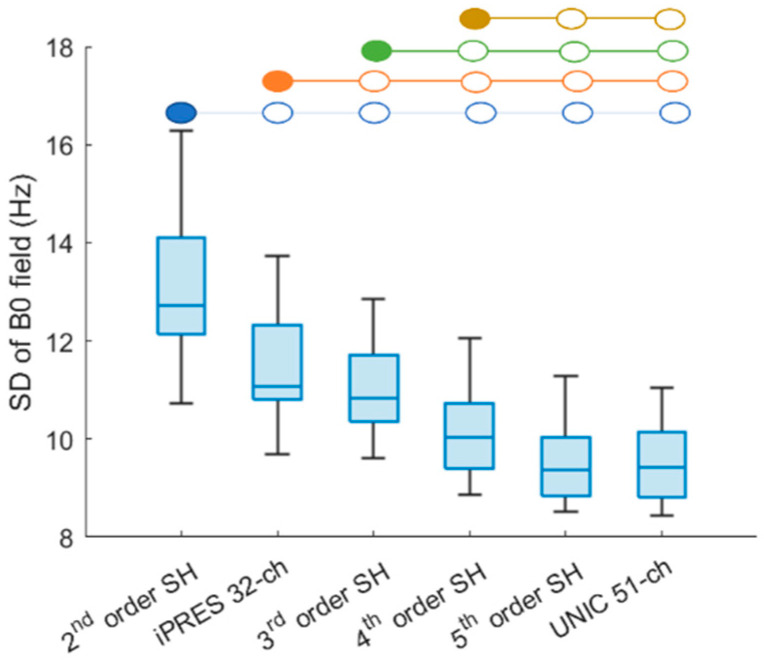
Box plot illustrating the standard deviation of frequency for 24 subjects in global shimming of the whole brain. UNIC 51-ch coil shimming exhibits significantly lower field variations than 2nd–4th-order SH and iPRES 32-ch coil shimming and performs similarly to 5th-order SH shimming (no difference; *p* = 1). The blue-colored circles indicate significant differences (*p* < 0.001) between 2nd-order SH with 3rd–5th-order SH, iPRES, and UNIC shim methods. Similarly, orange-colored circles indicate significant differences (*p* < 0.001) between 3rd SH with 4–5th-order SH, iPRES 32-ch, and UNIC 51-ch shimming. Green-colored circles indicate significant differences (*p* < 0.001) between iPRES with 4th–5th-order SH and UNIC shimming. Yellow-colored circles indicate significant differences (*p* < 0.001) between 4th with 5th-order SH and UNIC shimming.

The SD of the B_0_ field within the whole-brain volume, excluding the skull, was computed for twenty-four subjects. The UNIC 51-ch coil and theoretical 5th-order SH shimming exhibited the lowest SD of B_0_ field as compared to 2nd–4th-order SH and iPRES 32-ch coil shimming, as shown in Figure 2. The SD of the B_0_ field after either the UNIC 51-ch coil or 5th-order SH shimming exhibited a significant (*p* < 0.001) reduction of 27% and 17%, compared to the standard 2nd-order scanner shim and iPRES coil shimming, respectively. There were no significant differences between the standard deviation of the B_0_ field after the UNIC 51-ch coil and 5th-order SH shimming (*p* = 1). Additionally, iPRES 32-ch shim significantly outperformed the standard 2nd-order scanner shim (*p* < 0.001) but was slightly inferior to the theoretical 3rd-order SH shim (*p* < 0.001) as shown in Figure 2 and *p*-values provided in Supplementary Table S4.

### 3.2. Whole-Brain Coverage of 3D-MRSI with SH Shim, iPRES, and UNIC Shim

**Figure 3:**
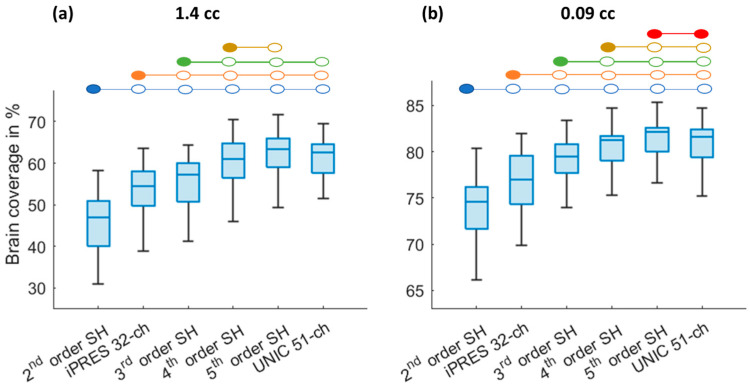
Box plots showing the comparison of brain coverage for MRSI at spatial resolutions (**a**) 1.44 cc and (**b**) 0.09 cc after shimming with theoretical (2nd–5th-order SH), iPRES, and UNIC shim. The blue-colored circles represent significant differences (*p* < 0.05) between 2nd-order SH with 3–5th-order SH, iPRES, and UNIC shim methods. Similarly, the orange-colored circles indicate significant differences between 3rd-order SH with 4–5th-order SH, iPRES, and UNIC shimming. Green-colored circles indicate significant differences between iPRES with 4–5th-order SH and UNIC shimming. Yellow-colored circles indicate significant differences between 4th-order SH with 5th-order SH and UNIC shimming. Red-colored circles indicate significant differences between 5th-order SH and UNIC 51-ch shimming.

Simulation of whole-brain 3D-MRSI coverage using SH shim and shimming with iPRES and UNIC shim coils were conducted. Brain coverage was commuted after shimming. Figure 3 shows a box plot illustrating brain coverages of 3D-MRSI with voxel sizes of 1.44 cc (Figure 3a) and 0.09 cc (Figure 3b) after 2nd–5th SH shimming, iPRES 32-ch, and proposed UNIC 51-ch shimming. For 3D-MRSI with a voxel size of 1.44 cc and isotropic resolution of 11.3 mm, UNIC 51-ch showed significant (*p* < 0.001) improvement in brain coverage of 61% compared to standard 2nd-order scanner shim (45%) and iPRES 32-ch shim (54%). More details about 2nd–5th-order SH shims are shown in Figure 3a and Table 1. Overall, UNIC 51-ch exhibited a 34% increase in the usable voxels as compared to the standard 2nd-order scanner shim and 13% compared to the iPRES coil shim. There was no significant difference between the brain coverage of UNIC 51-ch vs. 4th-order (*p* = 0.448) and the 5th-order (*p* = 0.679) SH shim. Additionally, the iPRES 32-ch shim performed significantly better than the standard 2nd-order scanner shim but was inferior to the theoretical 3rd-order SH shim.

For high-resolution 3D-MRSI with a voxel size of 0.09 cc and isotropic resolution of 4.5 mm, UNIC still provided significantly (*p* < 0.001) increased brain coverage by 9% as compared to the standard 2nd-order scanner shim, and 5% as compared to the iPRES shim. UNIC was significantly better than 2nd-, 3rd- (*p* < 0.001), and 4th (*p* = 0.004)-order SH for improving brain coverage. More details about 2nd–5th-order SH shims are shown in Figure 3b and Table 1, and *p*-values in Supplementary Table S5. Additionally, the iPRES 32-ch shim performs significantly better than the standard 2nd-order scanner shim but was significantly inferior to the theoretical 3rd-order SH shim.

### 3.3. Region-Specific Brain Coverage of 3D-MRSI with SH Shim, iPRES, and UNIC Shim

**Figure 4:**
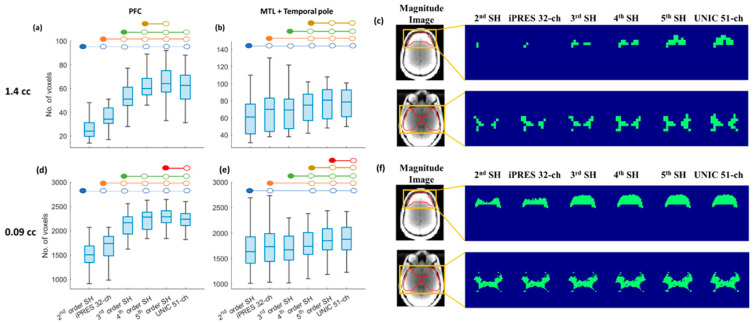
Box plot showing the comparison between brain coverage of MRSI resolution of (**a**–**c**) 1.44 cc and (**d**–**f**) 0.092 cc in (**a**,**d**) PFC and (**b**,**e**) MTL + temporal pole after shimming with 2nd–5th- order SH, iPRES, and UNIC shim. The blue-colored circles represent significant differences (*p* < 0.05) between 2nd-order SH with 3rd–5th-order SH, iPRES, and UNIC shim methods. Similarly, the orange-colored circle indicated significant differences between 3rd-order SH with 4th–5th-order SH, iPRES, and UNIC shimming. Green-colored circles indicate significant differences between iPRES with 4th–5th-order SH and UNIC shimming. Yellow-colored circles indicate significant differences between 4th-order SH with 5th-order SH and UNIC shimming. Red-colored circles indicate significant differences between 5th SH and UNIC 51-ch shimming. (**c**,**f**) The representative slices showing brain coverage of MRSI at (**c**) 1.44 cc and (**f**) 0.09 cc in PFC (1st row) and MTL + temporal pole (2nd row) after SH, iPRES, and UNIC head coil shimming.

Region-specific brain coverage with 2nd–5th-order SH, iPRES 32-ch, and UNIC 51-ch shim was calculated for B_0_ inhomogeneity in most affected brain regions, such as PFC, MTL, and temporal pole in this study. In the PFC, brain coverage at 1.44 cc after UNIC shim showed a significant (*p* < 0.001) and dramatic improvement, with the number of usable voxels increased by 147% compared to the standard 2nd-order scanner shim and 79% compared to the iPRES 32-ch shim, as shown in Figure 4a. Similarly, the PFC coverage at 0.09 cc showed a significant (*p* < 0.001) and dramatic improvement, with the number of usable voxels increased by 47% compared to the standard 2nd-order scanner shim and 31% compared to the iPRES shim, as shown in Figure 4d. For both 1.44 cc and 0.09 cc, UNIC shim performed significantly better than 2nd and 3rd-order but had no significant differences vs. 4th-order SH for improving PFC coverage. In addition, B_0_ field maps with UNIC shim in the PFC region showed a significantly (*p* < 0.001) lower SD, reduced by 26–57% compared to 2nd–3rd-order SH shim and iPRES shim. *p*-values of comparison of the brain coverage in the prefrontal cortex (PFC) for different shimming techniques can be seen in Supplementary Table S6.

For the region of MTL + temporal pole, brain coverage at 1.44 cc after UNIC shim showed a significant (*p* < 0.001) improvement, with the number of usable voxels increased by 29% compared to the standard 2nd-order scanner shim, and 17% compared to the iPRES 32-ch shim, as shown in Figure 4b. It should be noted that there were no significant differences between 2nd-order SH and 3rd-order shim and no significant differences between UNIC and 5th-order shim. Similarly, the regional coverage at 0.092 cc after the UNIC shim has a significant (*p* < 0.001) improvement, with the number of usable voxels in the region increased by 13% compared to the standard 2nd-order scanner shim, and 10% compared to the iPRES 32-ch shim, as shown in Figure 4e. In addition, UNIC shim significantly (*p* < 0.05) reduced the frequency SD of the B_0_ field map by 15–20% as compared to 2nd–4th-order SH and iPRES shim. *p*-values of comparison of the brain coverage in MTL + temporal pole for different shimming techniques can be seen in Supplementary Table S7.

In Figure 4f, a representative brain slice shows larger brain coverage at 1.44 cc in the PFC region (1st row of Figure 4c) after UNIC shimming, whereas there are very few usable voxels present after 2nd–4th-order SH shim or iPRES shim. Similar brain coverage improvement can be seen in the MTL + temporal pole region (2nd row of Figure 4c). For 0.09 cc resolution (Figure 4c), a representative brain slice shows larger brain coverage in the PFC region (1st row of Figure 4c) after UNIC shimming, whereas there are much fewer usable voxels present after 2nd-, 3rd-order SH shim or iPRES shim. Similar brain coverage improvement in the PFC can be seen for 4th- and 5th-order SH or UNIC shimming. In the MTL + temporal pole region (2nd row of Figure 4c,f), brain coverage improvement is also enhanced in the temporal pole region after UNIC shimming compared to 2nd–5th-order SH shimming.

**Figure 5:**
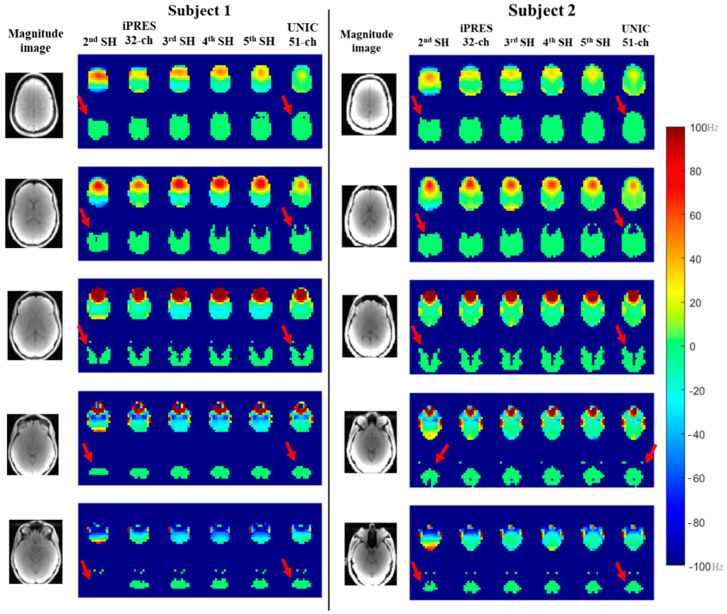
Representative slices of whole brain showing improved brain coverage of 3D-MRSI at 1.44 cc using the empirical UNIC head coil in two healthy subjects, in comparison with SH shimming (2nd–4th-order) and iPRES coil and showing similar brain coverage after 5th-order SH shimming. The red arrow indicates the improvement in brain coverage after UNIC shimming compared to standard 2nd-order scanner shim.

**Figure 6:**
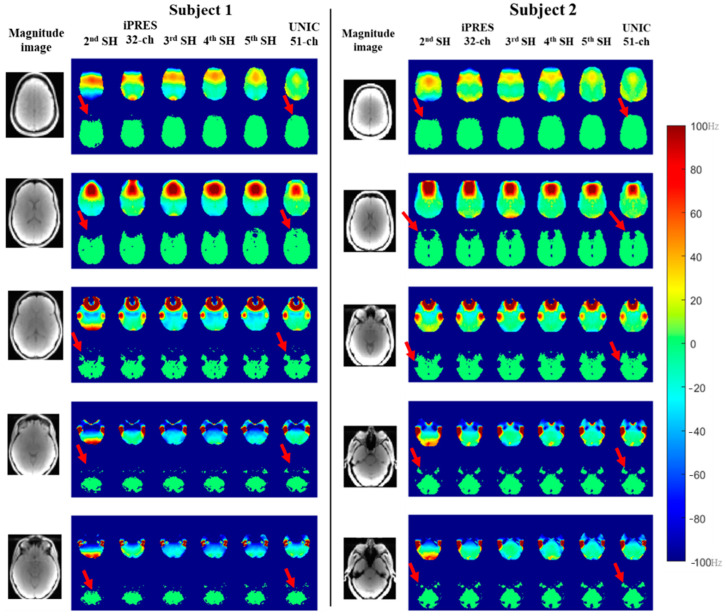
Representative slices of the whole brain showing improved brain coverage of 3D-MRSI at 0.092 cc using the empirical UNIC in two healthy subjects, in comparison with SH shimming (2nd and 3rd-order) and iPRES coil, showing similar brain coverage after 4th-order SH shimming. The red arrow indicates improvement in brain coverage after UNIC shimming compared to 2nd-order SH shim.

Representative slices shown in Figure 5 and Figure 6 demonstrate an improvement in B_0_ field and whole-brain coverage in MRSI at 1.44 cc and 0.09 cc with UNIC as compared to 2nd–5th-order SH shim and iPRES shim coils, respectively.

## 4. Discussions

Higher-order spherical harmonic (SH) shim coils, such as 3rd-, 4th-, and 5th-order SH shims, are not typically equipped in clinical scanners operating at 1.5T and 3T due to physical limitations. The incorporation of these higher-order SH shims necessitates the addition of 7, 9, and 11 concentric coils for each order, respectively, consuming valuable radial space within the MRI bore. Additionally, the higher-order shim coils require significantly increased currents, leading to substantial heating issues that cannot be effectively mitigated with in-bore water cooling.

Previous designs involving local coil shimming, such as using oral shim coils [39] and a separate local shim array [12,34,40], required placing small shim coils into the subject’s mouth, around the neck, or onto the face. These approaches may potentially cause patient discomfort and pose challenges for seamless integration into routine clinical workflow.

Our investigation utilized acquired brain field maps to demonstrate the effectiveness of the unified shim–RF coil in enhancing both whole-brain and region-specific coverage in 3D-MRSI. The empirical 51-ch UNIC design significantly improved brain coverage compared to the conventional 2nd-order scanner shim and 32-ch iPRES coil. Notably, the UNIC’s performance aligns with that achieved by the 5th-order SH shim, with significant enhancements observed within regions traditionally challenging for shimming, such as the prefrontal cortex, the temporal pole, and MTL.

The superior performance of the UNIC 51-ch coil is not merely due to an increased number of coils but primarily arises from the strategic arrangement of a greater number of RF-decoupled size-matched shim coils (4–5 cm) near the B_0_ inhomogeneity in most affected areas. This arrangement enables the shim coils to generate opposing high-order fields, effectively counterbalancing the inhomogeneous field in those regions. In contrast, iPRES is limited by RF-shim shared loops, resulting in over 9 cm diameter of loops in a standard 32-ch head coil to cover the brain. While modified iPRES versions [41] use smaller loops, the single turn of the RF loop remains challenging for generating sufficient field strength to shim deeper organs, such as the medial temporal lobe. On the other hand, UNIC can utilize multiple turn loops, significantly multiplying the shim field strength.

The UNIC demonstrated higher whole-brain coverage in comparison to the standard 2nd-order scanner shim for both low resolution (1.44 cc) and high resolution (0.092 cc) MRSI. This finding is particularly significant in scenarios characterized by lower spatial resolution, which typically leads to more severe intra-voxel dephasing or in instances where vendor-provided shimming solutions perform suboptimally. In the study by Sabati et al. [11], which assessed brain coverage of whole-brain MRSI acquired using three different MR vendors, it was revealed that one of the vendors achieved only 52% brain coverage of MRSI at a spatial resolution of 1.5 cc, with a wide range of 37% to 61%. In our study, MRSI with a resolution of 1.4 cc and UNIC shim demonstrated a remarkable 15% increase in whole-brain coverage. This leads to a one-third increase in usable voxels, particularly beneficial under suboptimal vendor shimming conditions.

The variability in shimming effectiveness between patients is another significant limitation of 3D-MRSI. However, with UNIC, it is noteworthy that the inter-subject variations in shimming effectiveness and brain coverage are markedly reduced while simultaneously increasing brain coverage. This observation aligns with the decreased variations in the SD of the off-resonance frequency distribution across different subjects.

A region-specific comparison was also conducted in this study. UNIC shimming demonstrated the greatest coverage increase in the PFC over the scanner shim by 147% and 50%, with 3D-MRSI voxel resolutions of 1.44 cc and 0.09 cc, respectively. This enhancement could prove invaluable in the investigation of psychiatric disorders, such as major depressive disorders (MDD) [42,43].

In this study, we observed that there were greater brain coverage improvements in the PFC region as compared to the temporal lobe. One of the reasons for this difference could be the more challenging shim requirements in the temporal lobe due to its complex structure. Secondly, the current shim algorithm, which prioritizes minimizing the frequency SD across the entire brain, consequently emphasizes shimming in the PFC to reduce the total SD of the brain, potentially at the expense of the temporal lobe. Future research would benefit from exploring algorithms that specifically address the shimming needs of the temporal lobe. This can ensure a more balanced and efficient shimming performance across all brain regions.

The empirical design of the 51-ch shim UNIC head coil applies evenly distributed large shim loops for whole-brain coverage and eight evenly distributed smaller shim loops targeted at the frontal and temporal lobes, respectively. This arrangement is advantageous in accommodating variable subject positions and anatomical variations. However, the presence of coil redundancy is evident, prompting ongoing efforts to reduce this via dimensionality reduction methods such as Principal Component Analysis (PCA) analysis [44]. While an increased number of shim coils generally translates to enhanced shimming performance, our decision to utilize 51 coils sought to balance between robust shim with hardware simplicity. Future designs aim to maintain or reduce the number of shim coils while optimizing coil arrangement to achieve higher-order shim capabilities, such as 7th- or 8th-order SH, and further increase brain coverage.

One limitation of the study is the simplistic approach to estimate of linewidth based on the off-resonance frequency SD for each voxel derived from the acquired brain field maps. This approach may not provide the highest level of accuracy. For future investigations, a more precise spectral fitting model could be implemented. However, it is important to consider the interplay of various experimental factors, such as subject motion and lipid signals, in the context of this study. The primary focus will be on prototyping the empirical UNIC head coil and acquiring realistic 3D-MRSI data. Experimental validation will be necessary, and the acquired data will play a crucial role in guiding further design improvements. Our focus on healthy subjects in this study sets the stage for future research to include neoplastic patients, assessed by two independent readers, to comprehensively validate the technology. Such validation is crucial for the potential clinical application of this technology in oncology, promising significant advancements in cancer diagnostics and treatment planning. Lastly, to fully evaluate the impact of UNIC shimming on MRSI in terms of signal quality, whole-brain coverage, and inter-rater reliability, a multi-institutional study with a large sample of subjects should be performed. This approach will test the repeatability and reproducibility of our proposed method, offering invaluable insights for future technological developments.

## 5. Conclusions

The more widespread clinical adoption of 3D-MRSI faces challenges related to magnetic field homogeneity across the brain and inter-patient variability in shimming. To address these challenges, we introduced a novel head coil design, the Unified Shim–RF coil (UNIC), aimed at improving whole-brain 3D-MRSI. Our findings indicate that the UNIC improves brain coverage and field homogeneity for both low (1.44 cc) and high resolution (0.09 cc) 3D-MRSI, surpassing standard 2nd-order scanner shimming and the iPRES shim–RF head coil. Moreover, UNIC reduces inter-subject variability in shimming quality and brain coverage, outperforming scanner shimming and iPRES shimming. Empirical UNIC design performs comparably to theoretical 5th-order spherical harmonic shimming. Future optimized designs, employing dimensionality reduction techniques, may achieve higher-order (over 6th-order) shimming. In summary, UNIC shows potential for enabling the more accurate quantification of metabolites in the entire brain with 3D-MRSI.

## Figures and Tables

**Table 1:** Brain coverage of 3D-MRSI at spatial resolutions of 1.44 cc and 0.09 cc with 2nd–5th-order SH, iPRES 32-ch, and UNIC 51-ch coil shimming techniques.

B_0_ Field Shimming Methods	1.44 cc ƚ	0.09 cc ƚ
**2nd-order SH**	45 ± 7	74 ± 4
**iPRES 32-ch**	54 ± 6	77 ± 3
**3rd-order SH**	56 ± 6	79 ± 3
**4th-order SH**	60 ± 6	80 ± 3
**5th-order SH**	62 ± 6	81 ± 3
**UNIC 51-ch**	61 ± 6	81 ± 3

ƚ Data represented in mean ± SD and %.

## Data Availability

The datasets presented in this study can be found in online repositories in HCP Connectome Central Facility: https://www.humanconnectome.org/study/hcp-young-adult/data-releases, accessed on 8 September 2022.

## Supplementary Materials

The following supporting information can be downloaded at https://www.mdpi.com/article/10.3390/cancers16061233/s1. Supplementary Table S1: X-, Y-, and Z-coordinates of 51 channel UNIC head coil loop position across the brain surface; Supplementary Table S2: Absolute mean and maximum current used in UNIC 51 channel head coils across twenty-four subjects; Supplementary Table S3: Coil currents in each 51 channel UNIC head coils in three representative subjects; Supplementary Table S4: *p*-values for Figure 2 showing the comparison of standard deviation of the frequency for 24 subjects in global shimming of the whole brain; Supplementary Table S5: *p*-values for Figure 3 showing the comparison of brain coverage for MRSI at spatial resolutions (a) 1.44 cc and (b) 0.09 cc; Supplementary Table S6: *p*-values for Figure 4a,d showing the comparison of brain coverage in prefrontal cortex (PFC) for MRSI at spatial resolutions (a) 1.44 cc and (b) 0.09 cc; Supplementary Table S7: *p*-values for Figure 4b,e showing the comparison of brain coverage in medial temporal lobe (MTL) + temporal pole for MRSI at spatial resolutions (a) 1.44 cc and (b) 0.09 cc.
