# Supplementary material for: Enhancing Whole-Brain Magnetic Field Homogeneity for 3D-Magnetic Resonance Spectroscopic Imaging with a Novel Unified Coil: A Preliminary Study
Source: Cancers (Basel). 2024 Mar 21;16(6):1233. doi: 10.3390/cancers16061233 (PMC10969602; doi:10.3390/cancers16061233)
Supplement: Supplementary file 1 [file cancers-16-01233-s001.zip › cancers-2892308-supplementary.pdf]

Supplementary material

Supplementary Table S1: X, Y, and Z-coordinates of 51 channel UNIC head coil loop position across the brain surface

Coil number	X (m)	Y (m)	Z (m)
**Coil 1**	0.04	0	0.1
**Coil 2**	-0.04	0	0.1
**Coil 3**	-0.098	-0.005	0.05
**Coil 4**	-0.068	0.051	0.07
**Coil 5**	0.068	0.051	0.07
**Coil 6**	0.098	-0.005	0.05
**Coil 7**	-0.089	0.063	0.016
**Coil 8**	-0.034	0.098	0.036
**Coil 9**	0.034	0.098	0.036
**Coil 10**	0.089	0.063	0.016
**Coil 11**	0	-0.07	-0.11
**Coil 12**	0	-0.021	-0.118
**Coil 13**	0	0.044	-0.112
**Coil 14**	0	0.096	-0.072
**Coil 15**	0	0.12	-0.01
**Coil 16**	-0.062	0.098	-0.03
**Coil 17**	-0.057	0.06	-0.087
**Coil 18**	-0.061	0.01	-0.103
**Coil 19**	-0.056	-0.05	-0.106
**Coil 20**	0.062	0.098	-0.03
**Coil 21**	0.057	0.06	-0.087
**Coil 22**	0.061	0.01	-0.103
**Coil 23**	0.056	-0.05	-0.106
**Coil 24**	-0.089	-0.024	-0.071
**Coil 25**	-0.08	0.045	-0.049
**Coil 26**	0.089	-0.024	-0.071
**Coil 27**	0.08	0.045	-0.049
**Coil 28**	0.02	0.047	0.109
**Coil 29**	-0.02	0.047	0.109
**Coil 30**	0.02	0.075	0.092
**Coil 31**	-0.02	0.075	0.092
**Coil 32**	0.02	0.097	0.068
**Coil 33**	-0.02	0.097	0.068

**Coil 34**	0.02	0.112	0.039
**Coil 35**	-0.02	0.112	0.039
**Coil 36**	-0.109	-0.04	0.015
**Coil 37**	-0.109	-0.01	0.015
**Coil 38**	-0.109	0.02	0.015
**Coil 39**	-0.102	0.048	0.014
**Coil 40**	-0.109	-0.04	-0.015
**Coil 41**	-0.109	-0.01	-0.015
**Coil 42**	-0.109	0.02	-0.015
**Coil 43**	-0.102	0.048	-0.014
**Coil 44**	0.109	-0.04	0.015
**Coil 45**	0.109	-0.01	0.015
**Coil 46**	0.109	0.02	0.015
**Coil 47**	0.102	0.048	0.014
**Coil 48**	0.109	-0.04	-0.015
**Coil 49**	0.109	-0.01	-0.015
**Coil 50**	0.109	0.02	-0.015
**Coil 51**	0.102	0.048	-0.014

Supplementary Table S2: Absolute mean and maximum current used in UNIC 51 channel head coils across twenty-four subjects.

**Absolute mean (Amperes)**	**Max (Amperes)**
0.41	2.31

Supplementary Table S3: Coil currents in each 51 channel UNIC head coils in three representative subjects

**Coil current**	**Subject 1**	**Subject 2**	**Subject 3**
**Coil 1**	-0.7394	-0.5662	-1.0458
**Coil 2**	-0.7494	-0.607	-0.8513
**Coil 3**	0.34509	-0.147	0.71077
**Coil 4**	-0.2209	-0.3687	0.0002
**Coil 5**	-0.2502	-0.2137	-0.07
**Coil 6**	0.2104	0.14722	0.83482
**Coil 7**	0.59142	0.4133	0.57737
**Coil 8**	0.06936	-0.0552	-0.0059
**Coil 9**	0.05847	-0.0472	0.03864
**Coil 10**	0.56465	0.40626	0.67413
**Coil 11**	0.01738	0.18101	0.02572

**Coil 12**	0.02616	0.14609	-0.0115
**Coil 13**	-0.0393	0.07656	-0.0347
**Coil 14**	0.05044	0.10302	0.04424
**Coil 15**	0.08522	0.04603	0.08379
**Coil 16**	0.15555	0.13871	0.07474
**Coil 17**	0.00329	0.04299	-0.0594
**Coil 18**	-0.0334	0.05341	-0.0955
**Coil 19**	-0.037	0.00416	0.03387
**Coil 20**	0.14319	0.10475	0.05357
**Coil 21**	0.02507	0.06174	0.00392
**Coil 22**	-0.0171	0.08419	-0.0087
**Coil 23**	-0.0296	0.04916	0.09768
**Coil 24**	-0.0335	0.02244	-0.1069
**Coil 25**	0.00067	0.01359	0.00507
**Coil 26**	-0.0192	-0.0265	-0.0432
**Coil 27**	0.00044	0.00829	0.00283
**Coil 28**	-0.1803	-0.2495	-0.4591
**Coil 29**	-0.3829	-0.177	-0.5517
**Coil 30**	-0.2902	-0.1341	-0.3369
**Coil 31**	-0.1865	-0.1635	-0.2894
**Coil 32**	-0.0093	-0.0588	-0.103
**Coil 33**	-0.1005	-0.0526	-0.1423
**Coil 34**	-0.0947	-0.0111	-0.0565
**Coil 35**	-0.0707	-0.029	0.0117
**Coil 36**	0.21596	-0.9395	-0.447
**Coil 37**	1.21245	1.32372	0.94338
**Coil 38**	0.67865	0.66394	0.26424
**Coil 39**	0.62409	0.31509	0.32838
**Coil 40**	1.20568	2.1337	1.68959
**Coil 41**	-0.1351	-0.2604	-0.2207
**Coil 42**	0.1308	0.21302	0.50056
**Coil 43**	-0.0387	0.14187	-0.0199
**Coil 44**	0.45667	-1.4838	-0.4372
**Coil 45**	1.09762	1.05078	1.21283
**Coil 46**	0.89135	0.60793	0.43705
**Coil 47**	0.65593	0.18239	0.39752
**Coil 48**	1.13982	2.68984	1.77637
**Coil 49**	-0.1187	-0.4153	-0.6145
**Coil 50**	0.06073	0.3007	0.45866
**Coil 51**	-0.0333	0.09149	-0.2373

Supplementary Table S4: P-values for figure 2 showing the comparison of standard deviation of the frequency for 24 subjects in global shimming of the whole brain.

B0 field shimming	2nd order SH	iPRES 32-ch	3rd order SH	4th order SH	5th order SH
iPRES 32-ch	p<0.001	-	-	-	-
3rd order SH	p<0.001	P=0.02	-	-	-
4th order SH	p<0.001	p<0.001	p<0.001	-	-
5th order SH	p<0.001	p<0.001	p<0.001	p<0.001	-
UNIC 51-ch	p<0.001	p<0.001	p<0.001	p<0.001	1

Supplementary Table S5: P-values for figure 3 showing the comparison of brain coverage for MRSI at spatial resolutions (a) 1.44 cc and (b) 0.09 cc.

B0 field shimming	2nd order SH	iPRES 32-ch	3rd order SH	4th order SH	5th order SH
**(a) 1.44 cc**					
iPRES 32-ch	p<0.001	-	-	-	-
3rd order SH	p<0.001	p<0.001	-	-	-
4th order SH	p<0.001	p<0.001	p<0.001	-	-
5th order SH	p<0.001	p<0.001	p<0.001	p<0.001	-
UNIC 51-ch	p<0.001	p<0.001	p<0.001	0.448	0.679
**(b) 0.09 cc**					
iPRES 32-ch	p<0.001	-	-	-	-
3rd order SH	p<0.001	p<0.001	-	-	-
4th order SH	p<0.001	p<0.001	p<0.001	-	-
5th order SH	p<0.001	p<0.001	p<0.001	p<0.001	-
UNIC 51-ch	p<0.001	p<0.001	p<0.001	0.004	0.02

Supplementary Table S6: P-values for figure 4 (a,d) showing the comparison of brain coverage in prefrontal cortex (PFC) for MRSI at spatial resolutions (a) 1.44 cc and (b) 0.09 cc.

B0 field shimming	2nd order SH	iPRES 32-ch	3rd order SH	4th order SH	5th order SH
**(a) 1.44 cc**					
iPRES 32-ch	p<0.001	-	-	-	-
3rd order SH	p<0.001	p<0.001	-	-	-
4th order SH	p<0.001	p<0.001	p<0.001	-	-
5th order SH	p<0.001	p<0.001	p<0.001	0.111	-
UNIC 51-ch	p<0.001	p<0.001	0.036	1	p<0.001
**(b) 0.09cc**					
iPRES 32-ch	p<0.001	-	-	-	-
3rd order SH	p<0.001	p<0.001	-	-	-
4th order SH	p<0.001	p<0.001	p<0.001	-	-
5th order SH	p<0.001	p<0.001	p<0.001	0.039	-
UNIC 51-ch	p<0.001	p<0.001	p<0.001	1	0.199

Supplementary Table S7: P-values for figure 4 (b,e) showing the comparison of brain coverage in medial temporal lobe (MTL) + temporal pole for MRSI at spatial resolutions (a) 1.44 cc and (b) 0.09 cc.

B0 field shimming	2nd order SH	iPRES 32-ch	3rd order SH	4th order SH	5th order SH
**(a) 1.44 cc**					
iPRES 32-ch	0.001	-	-	-	-
3rd order SH	0.593	1	-	-	-
4th order SH	p<0.001	0.002	p<0.001	-	-
5th order SH	p<0.001	p<0.001	p<0.001	p<0.001	-
UNIC 51-ch	p<0.001	p<0.001	p<0.001	p<0.001	0.035
**(b) 0.09cc**					
iPRES 32-ch	p<0.001	-	-	-	-
3rd order SH	p<0.001	1	-	-	-
4th order SH	p<0.001	p<0.001	p<0.001	-	-
5th order SH	p<0.001	p<0.001	p<0.001	p<0.001	-
UNIC 51-ch	p<0.001	p<0.001	p<0.001	p<0.001	1
